# Land Surface Model Calibration Using Satellite Remote Sensing Data

**DOI:** 10.3390/s23041848

**Published:** 2023-02-07

**Authors:** Mehdi Khaki

**Affiliations:** School of Engineering, University of Newcastle, Callaghan, NSW 2308, Australia; mehdi.khaki@newcastle.edu.au

**Keywords:** model calibration, satellite remote sensing, optimisation algorithm, terrestrial water storage, soil moisture

## Abstract

Satellite remote sensing provides a unique opportunity for calibrating land surface models due to their direct measurements of various hydrological variables as well as extensive spatial and temporal coverage. This study aims to apply terrestrial water storage (TWS) estimated from the gravity recovery and climate experiment (GRACE) mission as well as soil moisture products from advanced microwave scanning radiometer–earth observing system (AMSR-E) to calibrate a land surface model using multi-objective evolutionary algorithms. For this purpose, the non-dominated sorting genetic algorithm (NSGA) is used to improve the model’s parameters. The calibration is carried out for the period of two years 2003 and 2010 (calibration period) in Australia, and the impact is further monitored over 2011 (forecasting period). A new combined objective function based on the observations’ uncertainty is developed to efficiently improve the model parameters for a consistent and reliable forecasting skill. According to the evaluation of the results against independent measurements, it is found that the calibrated model parameters lead to better model simulations both in the calibration and forecasting period.

## 1. Introduction

Land surface models are valuable tools for simulating water resources and their variations over large scales and a long period of time. Furthermore, the models are important for simulating hydrological processes and forecasting their short- to long-term changes in different spatial and temporal resolutions. Nevertheless, due to various sources of errors, such as erroneous input and forcing data, imperfect modeling and (empirical) parameters, and limited datasets, models can face difficulties in realistically simulating various water components [1,2,3].

While using additional data through empirical or statistical techniques such as nudging or data assimilation scheme can improve the models’ performance, e.g., [4,5,6,7,8,9], the ability of the methods to enhance the model’s forecasting skill is questionable. The positive impact of these processes is largely limited to the period in which the data integration is carried out. A potential reason for this could be that only updating the model states without adjusting their parameters limits the ability of the system to maintain the improvements induced by the new data, e.g., [10,11,12]. Imperfect model parameters, if not adjusted regularly, e.g., using empirical or numerical methods, can limit the model’s ability to accurately perform simulations. This can be even more problematic during the forecasting period when larger uncertainties may exist in the model inputs. Thus, updating the model parameters for improved simulating/forecasting is critical. This, however, can be more challenging than updating the model states since the parameters are usually not being measured directly. As a result, different parameter calibration schemes have been put forward to use independent observations to estimate parameters during the data integration process e.g., [13,14,15,16].

The model calibration process adjusts the parameters such that the simulations from the model match observations, e.g., from remote sensing as closely as possible [13]. In this context, automatic optimisation techniques are developed, e.g., [14,15] with the main objective of exploring the best model parameters that lead to a minimum discrepancy between the model simulation and observed data. The approach has been successfully applied in different cases. For example, [17] applied the parameter optimisation to enhance the dynamic vegetation module of the Noah model over the Chinese Loess Plateau. The model calibration via parameter optimisation techniques has also been carried out to improve the modeling of water and carbon fluxes from land surface models, e.g., [18,19]. The optimisation process is usually carried out using one or more goodness-of-fit numerical measures such as maximizing or minimizing an objective function [16]. For the purpose of parameter calibration using additional data, one single-objective function such as the Nash–Sutcliffe efficiency (NSE) coefficient can be utilised. Nevertheless, as argued by [20], it can often be difficult to fit a majority of simulations to the observations relying on merely a single-objective function. To address this, multi-objective calibration has been put forward that utilises a combination of multiple single-objective functions with different associated weights that are defined by users [21]. The technique was recently used by [22] for parameter calibrations using a combination of satellite remote sensing data to achieve high accuracy runoff simulation.

The main goal of this effort is to apply a multi-objective calibration technique to adjust the parameters of a land surface model using satellite remotely sensed datasets. An optimisation method along with multiple observations of terrestrial water storage (TWS) data derived from the gravity recovery and climate experiment (GRACE), as well as soil moisture products retrieved from advanced microwave scanning radiometer–earth observing system (AMSR-E) are used to calibrate a land surface model. The optimisation algorithm has been tested widely in hydrology, particularly for rainfall-runoff applications, e.g., [13,23,24,25,26,27,28]. However, to the best of the author’s knowledge, this approach has not yet been fully investigated when integrating TWS and soil moisture simultaneously when a distributed land surface model is assumed. This can be a challenging task due to the models’ complexity and a larger number of parameters involved in the process. A few previous studies have analyzed the potential impact of the parameter optimisation on land surface models and reported considerable improvements in the simulations, e.g., [17,18,19,29]. This is usually achieved by relying on ground measurements, over limited spatial and temporal scales. This study, however, explores this further by using multiple satellite remote sensing data over various regions.

Furthermore, contrary to many previous studies, a multiple-objective function approach is implemented to achieve better estimates by more accurately constraining the outputs. The calibration is carried out using the multi-objective evolutionary algorithm of the non-dominated sorting genetic algorithm (NSGA) [30]. The approach, which has been applied in various hydrological applications, e.g., [30,31], is based on a genetic algorithm utilizing modified mating and survival selection. It works with a population of fixed size and compares individuals according to a sorting system to select the best individuals as the new parent population [31]. The GRACE-derived TWS and satellite soil-moisture observations along with their associated errors are used to optimise the model parameters while assessing the capability of the NSGA approach. A more realistic optimisation process needs to take measurement uncertainties into account. This is carried out here by incorporating uncertainty terms into the objective functions.

## 2. Dataset

### 2.1. Satellite Remote Sensing

TWS changes and soil moisture data are derived from GRACE and AMSR-E satellites, respectively. For the former, GRACE level 2 (L2) Stokes’ and their error coefficients (to degree 90) are obtained from the ITSG-Grace2014 [32]. These are converted to TWS changes between 2003 and 2011 following [33] using multiple standard steps. The TWS changes are used to assess the summation of model simulations including water stored in different soil layers, groundwater, and surface storage [8]. AMSR-E soil-moisture observations are collected over the same period of 2003–2011 and used during optimisation to assess the model simulations of surface soil moisture. Error standard deviations of 10 (mm) and 0.05 (m${}^{3}$ m${}^{-3}$) are assumed for the GRACE TWS changes and soil-moisture observation uncertainties, respectively, suggested by [34,35,36]. These are considered to be fixed for different grids and over time.

### 2.2. Land Surface Model

The world-wide water resources assessment (W3RA) land surface model, primarily developed for the Australian continent, is used for this experiment [37]. The W3RA model, which is a grid-distributed water balance system, however, simulates water storage and its flows globally. It generally simulates the balance between groundwater, surface water, and soil moisture independently at each grid cell [38,39]. This simulation is carried out separately for two hydrological response units (HRUs) occupying a fraction of each grid. These HRUs include tall and deep-rooted vegetation (HRU1) and short and shallow-rooted vegetation (HRU2). Correspondingly, parameterisations are applied on the sub-grid basis to simulate water compartments such as surface water and groundwater saturation at the area fractions [40,41]. Details of all of the parameters used here for optimisation are listed in Table 1. The initial values in the table show the corresponding parameter values that are used for the open-loop run (model run without calibration) and the model initialisation. The model’s forcing data consists of meteorological fields of maximum and minimum temperature, precipitation, and downwelling radiation selected here from the ERA-5 [42] daily reanalysis data for 2003–2011. This results in a daily model run for the same period at $0.5^{\circ }$ × $0.5^{\circ }$ spatial resolution over Australia as well as the Mississippi Basin (due to the ground data availability). It is worth mentioning that all observations are rescaled to $0.5^{\circ }$ × $0.5^{\circ }$ to match the model simulations during the calibration process.

### 2.3. In Situ Data

Two main types of in situ data are used to examine the performance of the implemented calibration approach. These include soil moisture and groundwater measurements derived over two river basins of the Mississippi Basin and the Murray–Darling Basin. These basins are chosen in this study subject to the availability of these measurements. The datasets are acquired from the United States Geological Survey (USGS) over the Mississippi Basin and from New South Wales Government (NSW; for groundwater) and the moisture-monitoring network (for soil moisture) over the Murray–Darling Basin.

## 3. Methodology

### 3.1. Sensitivity Analysis

In order to identify the more important parameters for calibration, a sensitivity analysis is applied that helps with measuring the model behaviour with respect to the parameter changes. This is carried out using a methodology developed by [43], entitled global sensitivity analysis. The approach helps to quantify the impact of the model parameters on its simulations. Contrary to local sensitivity techniques, the applied approach examines the model sensitivity over the whole parameter space. This approach is based on a variance technique that explores how each model’s individual parameter affects the full output variance. Assuming y from y=f(X) and $X=(x_{1},x_{2},\ldots ,x_{n})$ (n, the number of parameters), the goal in the global sensitivity analysis is to quantify the significance of parameters on the output variance, i.e., $S_{i}$ (sensitivity) of y to $x_{i}$ via,

(1) $$\begin{array}{c} S_{i}=\frac{var\{E\left[y|x_{i}\right]\}}{var\{y\}}. \end{array}$$

Important in Equation (1) is estimating the expected value of the output from an n-dimensional integral, which can be carried out numerically more efficiently using the Monte Carlo algorithm [44]. The method then focuses on the estimation of Sobol’ sensitivity indexes [45], which measure the effect of both parameters’ variation and their interaction (see details in [43]).

### 3.2. Optimisation Method

The main rational behind the optimisation algorithms in model calibration is to minimise the discrepancies between model simulations and a set of observations by adjusting the model parameters. To measure this departure from the observations, single- or multi-objective functions can be used. Both approaches look for the best solution corresponding to the maximum or minimum value of one (i.e., single-objective optimisation) or multiple (i.e., multi-objective optimisation) objective functions. Multi-objective function optimisation, contrary to the single-objective method, can better fit a majority of simulations to the observations [20]; thus, it is used here. It can also take into an account the interaction between various objectives, which results in a group of solutions [46]. The relationship between objective functions as in $F_{i}\left(\theta \right),i=1\ldots m$ with θ and *m* indicates the parameters and the number of functions, respectively, which can be described by

(2) $$\begin{array}{c} min[F_{1}\left(\theta \right),F_{2}\left(\theta \right),\ldots ,F_{m}\left(\theta \right)]. \end{array}$$

#### 3.2.1. Objective Functions

The objective functions here measure two major hydrological components of TWS and soil moisture (Sm) through two evaluation indexes, which are used to quantify the variables (both simulation and observation) goodness-of-fit. These indexes include Nash–Sutcliffe efficiency (NS) and root mean square error (RMSE), which are defined via,

(3) $$\begin{array}{c} F_{1}\left(\theta \right)=1-\frac{\sum_{i=1}^{N}\omega_{i}{\left(TWS_{i}^{obs}-TWS_{i}^{sim}\left(\theta \right)\right)}^{2}}{\sum_{i=1}^{N}{\left(TWS_{i}^{obs}-T\bar{W}S^{obs}\right)}^{2}}, \end{array}$$

(4) $$\begin{array}{c} F_{2}\left(\theta \right)={\left[\frac{1}{N}\sum_{i=1}^{N}\omega_{i}{\left(TWS_{i}^{obs}-TWS_{i}^{sim}\left(\theta \right)\right)}^{2}\right]}^{1/2}, \end{array}$$

(5) $$\begin{array}{c} F_{3}\left(\theta \right)=1-\frac{\sum_{i=1}^{N}\omega_{i}{\left(Sm_{i}^{obs}-Sm_{i}^{sim}\left(\theta \right)\right)}^{2}}{\sum_{i=1}^{N}\omega_{i}{\left(Sm_{i}^{obs}-S{\bar{m}}^{obs}\right)}^{2}}, \end{array}$$

where $F_{1}\left(\theta \right)$ and $F_{2}\left(\theta \right)$ refer to NS and RMSE between TWS from model simulations ($TWS^{sim}$) and GRACE ($TWS^{obs}$), respectively. $F_{3}\left(\theta \right)$ denotes NS between soil moisture (Sm) from model simulations ($Sm^{sim}$) and satellite remote sensing ($Sm^{obs}$). *N* in Equations (3)–(5) represents the dimension of simulated and observed data. Important in these questions is the addition of weighting coefficients, ω, which are an inverse form of observation uncertainties derived from observations, e.g., from GRACE in Equations (3) and (4) and satellite soil moisture in Equation (5). This allows for an optimum and realistic calibrations scheme, e.g., to avoid overemphasising observations where there is a large error.

To solve the combination of objective functions, Equation (2) can be simplified into one objective optimisation problem that aggregates all different objective functions, which is called Euclidean distance [16] and is formulated by

(6) $$\begin{array}{c} F\left(\theta \right)={\left[{\left(F_{1}\left(\theta \right)+A_{1}\right)}^{2}+{\left(F_{2}\left(\theta \right)+A_{2}\right)}^{2}+{\left(F_{3}\left(\theta \right)+A_{3}\right)}^{2}\right]}^{1/2}. \end{array}$$

$A_{1}$, $A_{2}$, and $A_{3}$ in Equation (6) are transformation constants of various objective functions, which are selected to weight equally as Equation (7) to prohibit excessive computational burden. This equation allows every single-objective function to posses the equal origin distance leading to equal weights for the various objectives [16].

(7) $$\begin{array}{c} A_{i}=max\left\{F_{j,min},j=1\ldots 3\right\}-F_{i,min},i=1\ldots 3. \end{array}$$

#### 3.2.2. Evolutionary Calibration Methods

The numerical evolutionary optimisation algorithm of NSGA [30] is used to yield an optimal solution. NSGA, first, uses a Pareto ranking mechanism to classify the solutions and density estimator (also known by crowding distance) to keep the diversity between the individual solutions [47]. This method searches to maintain the best members from the produced parameters (see more details in [30]).

## 4. Results

### 4.1. Sensitivity Analysis

**Figure 1:**
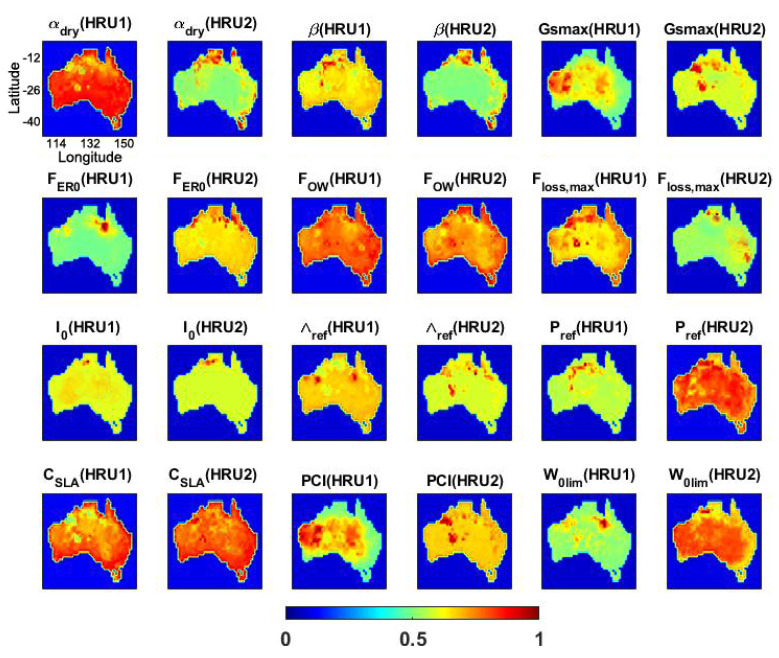
Average parameters’ sensitivity in each grid point over Australia.

**Figure 2:**
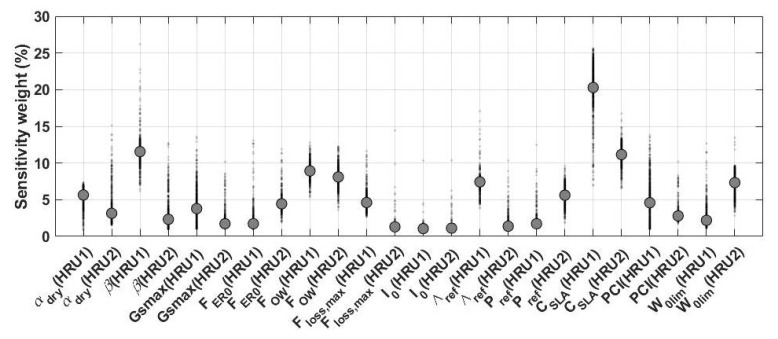
Percentage sensitivity of the model toward 24 selected parameters. Different shaded weights in the figure correspond to different samples for the Monte Carlo simulation. The average value of each parameter is presented by solid crosses.

The sensitivity analysis is carried out to measure the impact of the model’s parameters on its output. The results of the applied sensitivity analysis can further reveal the importance of the calibration process. Figure 1 shows the relative sensitivity of each tested parameter (cf. Table 1) within Australia. It can clearly be seen that the spatial variability is dominant for most of the parameters. This is particularly important due to the fact that the parameters are defined to be fixed spatially and temporally. The sensitivity analysis, however, demonstrates that such an assumption cannot be realistic, at least for a large group of parameters such as $\alpha_{dry}$, $C_{SLA}$, and PCI. A few parameters such as $I_{0}$ and, to a lesser degree, $F_{ER0}$ indicate fewer spatial changes and can be assumed to be constant over different areas. It can also be inferred from the figure that the level of model sensitivity to parameters is different. For example, the variation of $C_{SLA}$ and $F_{OW}$ is more sensitive than some other parameters, e.g., β, $I_{0}$, and $\Lambda_{ref}$. This can be better seen in Figure 2.

Figure 2 depicts the percentage sensitivity weight of different parameters. The different weights in the figure correspond to 100 different samples for the Monte Carlo simulation. These numbers of samples are found by trial and error to sufficiently represent the weights of the parameters. Similar to Figure 1’s results, the model is more sensitive to the variation of $C_{SLA}$, β, $F_{OW}$, and $W_{0lim}$ (mainly in HRU2 and short and shallow-rooted vegetation). Both figures stress the importance of parameter $C_{SLA}$, which has the highest weight. This suggests that the specific leaf area has a substantial effect on the model simulations. The parameter’s interaction with moisture (humidity) and light levels is also very important. The lowest weights and correspondingly the least sensitivity refers to $I_{0}$, $G_{smax}$, and $F_{ER0}$, mostly with fewer physical properties. From Figure 2, the sensitivity variabilities are not limited to different parameters or grid points (cf. Figure 1), but this also varies for different hydrological response units. These show the effect of the variations of the model parameter on the simulations that demonstrate the necessity of the meaningful selection of parameters for the calibration process.

### 4.2. Parameter Calibration

**Figure 3:**
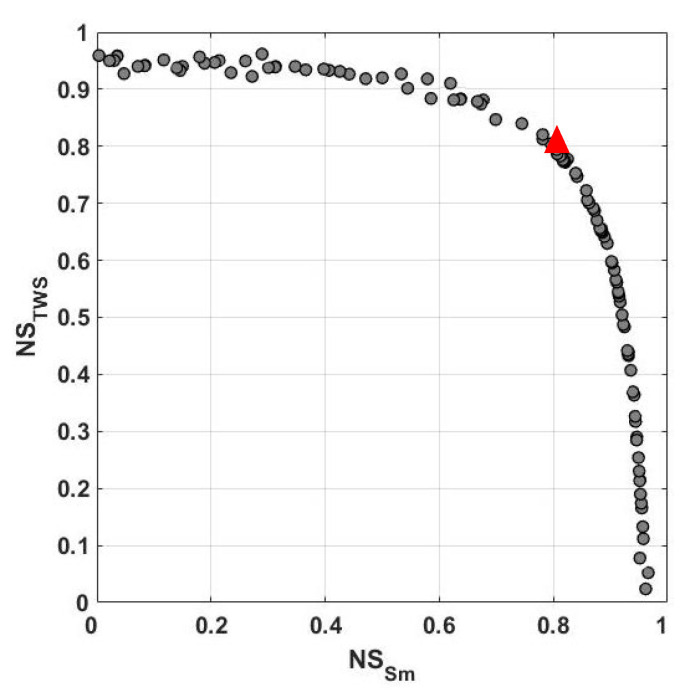
The average NS values with respect to GRACE TWS and satellite soil moisture used in optimisation objective functions. The selected optimum value, as a balance between NS derived from TWS and soil moisture, is represented by the red triangle.

**Figure 4:**
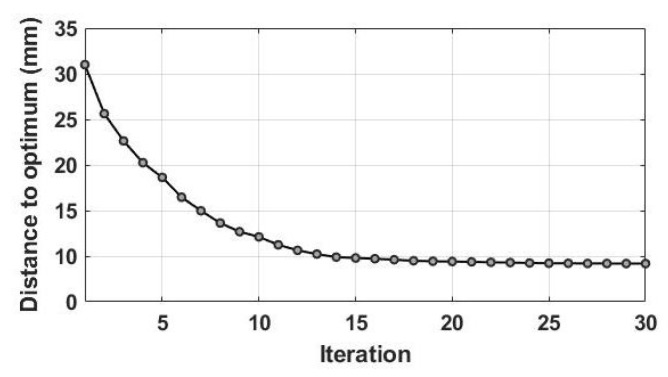
The distance to an optimum value, i.e., observations, in the first 30 iterations.

The parameter calibration results are assumed in this section. As explained in Section 3, the main tool for the algorithms to calibrate the parameters is using the objective function to control the deviation of the model simulations from observations as in GRACE TWS and satellite soil moisture. In order to show this, Figure 3 exemplifies the average NS values over the study area. These NS values are estimated using the applied method while implementing objective functions. Note that for a better illustration, only the results of NS functions are demonstrated. The optimum value is selected arbitrarily as a balance between NS derived from TWS and soil moisture and represented by red triangles in the figure. The optimum parameter corresponds to this selection. As is clear from Figure 3, high NS values are obtained from the implementation of NSGA for both TWS and soil-moisture tests. The approach shows small variations in NS values, which can be explained by its ability to achieve faster convergence. This can lead to selecting an optimum value that corresponds to higher NS estimates. To further investigate this, the average distance to an optimum value, i.e., observations, in the first 30 iterations is depicted in Figure 4. NSGA converges after 12 iterations, which shows that the method that performs reasonably well in solving for the objective function with high NS values (cf. Figure 3) can also reach optimum value in a small number of iterations by reducing the distance between the model simulations and observations.

**Figure 5:**
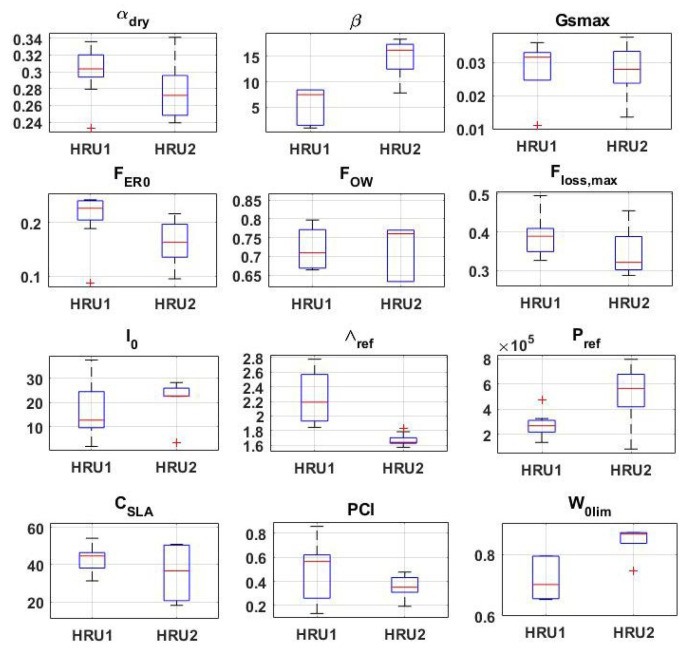
The adjusted parameters with their corresponding range of variations.

The adjusted parameters and their range of variations are presented in Figure 5. As can be seen, all parameters fall within the predefined ranges. While variations can be seen for all of the estimated parameters, some parameters indicate smaller variabilities such as $P_{ref}$ (HRU1) and $\Lambda_{ref}$ (HRU2). The parameters’ spread around the mean value is also different for various parameters. This non-uniform distribution can affect the parameters’ convergence. The distributions of the parameters can provide more information by analysing their kurtosis and skewness, which present the departure of the parameter distributions from Gaussian (with kurtosis 3 and skewness 0). Although following a Gaussian distribution does not guarantee better performance, it can show the method’s ability to successfully draw samples for each parameter. Parameters with smaller skewness and kurtosis are expected to better span the subspace of the model’s parameter [48,49]. Skewness measures the distribution asymmetry, and Kurtosis measures the distribution shape (i.e., light-tailed or heavy-tailed compared to Gaussian distribution) [50]. The average parameters skewness and kurtosis are calculated for all parameters. The average skewness and kurtosis-3 are found to be 0.13 and −1.05, respectively. The small skewness value, generally, indicates good asymmetry for NSGA. The negative kurtosis-3 means it has lighter tails and a flatter peak than the normal distribution as it can also be seen from Figure 5. These results show a reasonable distribution of parameters by NSGA, which, along with its good estimates of the objective function, can result in better parameter estimation. This is investigated by evaluating the model’s simulation using the adjusted parameters.

### 4.3. Results Assessment

**Figure 6:**
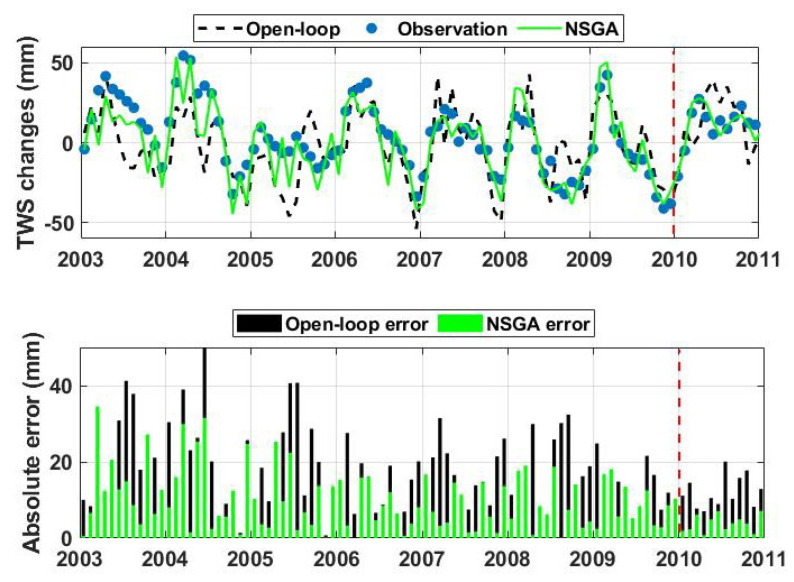
Average TWS variation from NSGA, model without calibration (open-loop), as well as observations over Australia (**top** panel). The corresponding errors, i.e., the difference between GRACE TWS and model simulation with and without calibration, is presented in the (**bottom** panel).

The performance of the optimisation algorithm for simulating water storage is assumed in this section separately for the calibration (2003–2010) and forecasting (2010–2011) periods. To start with, the time series of the average TWS changes from the model simulations with and without calibration (NSGA and open-loop, respectively), as well as GRACE, as shown in Figure 6 top panel. It can clearly be seen that the calibration process reduces the misfit between the model and observations in both experiment periods. The application of optimisation successfully reflects the observations changes, especially those more extreme ones such as the positive anomaly in 2003–2004. The TWS simulations after calibration also better represent observation trends and variations during 2010 (forecasting period). This model simulation improvement is evident from the error bars plotted in Figure 6 bottom panel, which represents the difference between GRACE TWS and the model simulations. Two average error values are calculated for each error bar, i.e., an average of the absolute difference between observation and, respectively, open-loop (black) and NSGA results (green). These are calculated separately for both the calibration and forecasting periods. The largest error reduction can be observed in the calibration period (from 18.61 mm to 8.55 mm). It can also be seen that the approach decreases errors compared to the open-loop run results in the forecasting period, i.e., from 9.27 mm to 4.80 mm. This improvement, however, is smaller, which can clearly be explained by the absence of the optimisation process.

**Figure 7:**
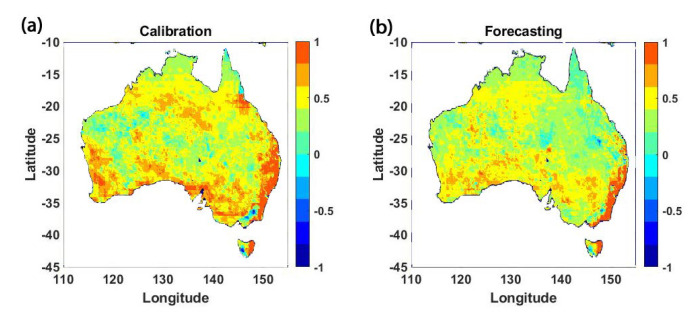
Correlation improvement by NSGA compared to the open-loop run with respect to the AMSR-E data. (**a**) shows the results for the calibration period (2003–2010), and (**b**) shows the results for the forecasting period (2010–2011).

In addition, the estimated soil moisture values from the open-loop run and NSGA results are compared with the AMSR-E data. To this end, first, correlation values are calculated at each grid point over Australia, and then the correlation improvements are computed and demonstrated in Figure 7. This is carried out by calculating the difference between the NSGA results correlations and those of the open-loop run, again separately for the calibration (2003–2010) and forecasting (2010–2011) periods. As can be seen from Figure 7, the implementation of the optimisation technique leads to correlation improvements over both periods. This, however, is more pronounced for the calibration period when the parameter is adjusted. Yet, a significant amount of the impact is maintained over the forecasting period, which shows the effectiveness of the applied approach for calibrating the model parameters.

**Figure 8:**
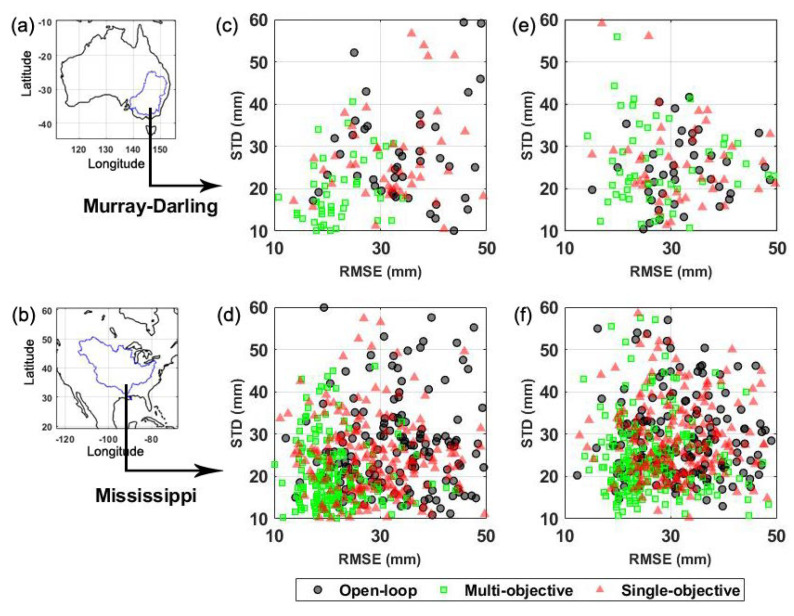
(**a**,**b**) show the location of the Murray–Darling and Mississippi basins, respectively. RMSE and STD values are calculated using the groundwater measurements (in situ) for those estimated with calibration using the multi-objective (green) and the single-objective (red) functions, as well as without (grey) calibration. (**c**,**d**) present the results for the calibration period over the Murray–Darling and Mississippi basins, respectively. (**e**,**f**) show the corresponding results for the forecasting period.

Groundwater in situ measurements over two basins of Murray–Darling and Mississippi are further used to evaluate the outcomes. The simulated groundwater changes are interpolated from different grid points to the nearest location of the observation bore. Afterwards, the RMSE and standard deviation (STD) values and their average from the groundwater measurements (in situ) and those estimates with and without calibration, as well as for the calibration and forecasting periods, are computed (Figure 8). All of the calibration results presented so far belong to the multi-objective function approach. To better investigate the performance of the parameter calibration scheme on the results, here, two different cases are assumed: (1) parameter calibration using a single-objective function (relying only on NS), and (2) parameter calibration using the multi-objective function (relying both on NS and RMSE).

Considerable improvements are obtained by the applied calibration methods, particularly for the calibration period (Figure 8c,d). A reduction in RMSE and STD can also be seen for the forecasting period for both cases (Figure 8e,f). The multi-objective function, however, obtains better results over both periods compared to the single-objective function, i.e., the average RMSE reduction of 40.54 mm against 14.10 mm for the calibration period and 20.17 mm against 5.98 mm for the forecasting period (over both basins). Noteworthily, the improvement in groundwater estimates is more pronounced over the Mississippi basin. The reason for this can be due to the fact that groundwater changes show larger variabilities over the Mississippi basin during the study period, which leads to larger discrepancies between the different methods’ results. In the Murray–Darling basin, this is more considerable for the calibration period, which can be explained by the fact that the parameter calibration method is actively being applied during this time. The multi-objective function successfully reduces STD values by 35% and 22% with respect to the open-loop for the calibration and forecasting periods, respectively. The achieved reductions for the single-objective function are 19% and 7% for the calibration and forecasting periods, which indicate the superiority of the multi-objective function case, while both cases outperform the open-loop run. Overall, the results showcase the usefulness of calibration optimisation, specifically using the multi-objective function for improving the model forecasts.

The performance of the calibration method is also examined with respect to the soil moisture from in situ measurements over both basins. Correlation analysis is used for this purpose. Estimated soil moisture from various model layers is compared with the corresponding in situ layers. For example, the simulated top layer soil moisture is compared with the top 8 cm and 10 cm measurements over the Murray–Darling and Mississippi basins. The average results are reported in Table 2. To test the significance of the correlation results, a significance test is implemented using t-distribution (0.05 significant level). In general, higher correlation values to the in situ measurements are achieved from the application of the optimisation methods, i.e., both single- and multi-objective functions compared to the open-loop run over both basins. Nevertheless, relatively higher correlation improvements are obtained during the calibration period, e.g., on average 1.96% and 2.1% higher for the single- and multi-objective functions. Particularly, the optimisation approach using the multi-objective function achieves better correlation values to the in situ measurements than the case with the single-objective function. From Table 2, the average correlation between the soil moisture estimates from the multi-objective function calibration over both basins and periods is 0.74, which is ∼4% higher than that of the single-objective function calibration. In general, NSGA, especially when using the multi-objective function, acquires improvements in different scenarios, i.e., different basins and different periods. The application of the optimisation algorithm, here using NSGA, can improve model soil moisture simulations and more importantly with a stable effect, which can last during the forecast period.

## 5. Conclusions

Land surface models are valuable tools for simulating and forecasting water compartments over land. However, the models can be subjected to various sources of errors, which make their adjustment necessary. Satellite remote sensing data are valuable tools to enhance the models and have attracted a lot of attention in the past two decades. The present study investigated the ability of remotely sensed data, including GRACE TWS and satellite soil moisture, to calibrate the land surface model using a multi-objective evolutionary algorithm of NSGA. This is carried out by calibrating the model’s parameters and reducing its simulation errors. Moreover, a new combined objective function based on the observations’ uncertainty is proposed to efficiently improve the model parameters for a consistent and reliable forecasting skill. Different characteristics of the method are explored, and it is found that the NSGA approach largely incorporates observations into the model estimates, leading to a faster calibration convergence to an optimum parameter value. It is also observed that the method can effectively improve the model simulations both in the calibration and forecasting period. The latter highly relies upon the improved parameters as a result of NSGA implementation. The assessment against independent soil moisture and groundwater measurements over the Mississippi and Murray–Darling basins further confirms this. It is shown that NSGA performs well in terms of improving the accuracy of the water storage simulations and forecasting skills. The approach leads to better groundwater and soil moisture simulations, not only during the calibration period but also in the forecasting period compared to the open-loop run.

## Figures and Tables

**Table 1:** The list of the W3RA model parameters along with their ranges [37].

Parameter	Range for HRU1	Range for HRU2	Initial Value (HRU1)	Initial Value (HRU2)	Parameter Description
$\alpha_{dry}$	[0.19–0.35]	[0.19–0.35]	0.26	0.26	Dry soil albedo
β	[0.70–8.40]	[0.70–18.40]	1.21	13.55	To determine the rate of hydraulic conductivity relevant to water content change
$C_{SLA}$	[0.70–71]	[0.70–71]	3	10	Specific leaf area per unit dry leaf biomass (unit: m${}^{2}$/kg)
PCI	[0.01–1.00]	[0.01–1.00]	0.052	0.069	Photosynthetic capacity index
Gsmax	[0.009–0.05]	[0.009–0.05]	0.03	0.03	Multiplier for deriving Gsmax from PCI (unit: m/s)
$F_{ER0}$	[0.05–0.25]	[0.05–0.25]	0.145	0.044	Wet canopy evaporation rate ratio with respect to rainfall rate
$F_{OW}$	[0.60–0.80]	[0.60–0.80]	0.7	0.7	Open water evaporation scaling factor
$F_{loss,max}$	[0.25–0.50]	[0.25–0.50]	0.3	0.3	Maximum fraction of net radiation (daytime) that is lost to heat storage in the absence of vegetation (-)
$I_{0}$	[0–41]	[0–41]	25.28	7.35	Initial retention capacity (unit: mm)
$\wedge_{ref}$	[1.30–3.50]	[1.30–2.50]	2.5	1.4	Reference leaf area index (LAI) to determine canopy cover
$P_{ref}$	[54–1,000,000]	[254–1,000,000]	148.1	732.6	Reference precipitation event for producing runoff (unit: mm/d)
$W_{0lim}$	[0.60–0.89]	[0.60–0.89]	0.85	0.85	Relative water content of top soil

**Table 2:** Average values of the calculated correlation between in situ and soil moisture (from the model) from various runs, as well as experiment periods. Note that improvements are calculated as [(Calibration − open-loop run)/open-loop run] × 100(%).

		Mississippi Basin		Murray–Darling Basin	
	Method	Calibration Period	Forecasting Period	Calibration Period	Forecasting Period
Model run cases	Open-loop	0.62	0.58	0.72	0.74
	Single-objective calibration	0.71	0.65	0.75	0.76
	Multi-objective calibration	0.75	0.67	0.75	0.78
Improvements (%)	Single-objective calibration	14.52	12.07	4.17	2.70
	Multi-objective calibration	20.97	15.52	4.17	5.41

## Data Availability

All the materials used in this study are listed in the paper. The W3RA model can be obtained from the Commonwealth Scientific and Industrial Research Organisation (CSIRO; http://www.wenfo.org/wald/data-software/, accessed on 16 January 2023).
